# Characterization of salt-tolerant plant growth-promoting rhizobacteria and the effect on growth and yield of saline-affected rice

**DOI:** 10.1371/journal.pone.0238537

**Published:** 2020-09-04

**Authors:** Rakiba Shultana, Ali Tan Kee Zuan, Mohd Rafii Yusop, Halimi Mohd Saud

**Affiliations:** 1 Department of Land Management, Faculty of Agriculture, Universiti Putra Malaysia, Serdang, Selangor, Malaysia; 2 Agronomy Division, Bangladesh Rice Research Institute, Gazipur, Bangladesh; 3 Department of Crop Science, Faculty of Agriculture, Universiti Putra Malaysia, Serdang, Selangor, Malaysia; 4 Institute of Tropical Agriculture and Food Security, Universiti Putra Malaysia, Serdang, Selangor, Malaysia; 5 Department of Agriculture Technology, Faculty of Agriculture, Universiti Putra Malaysia, Serdang, Selangor, Malaysia; Carnegie Mellon University, UNITED STATES

## Abstract

In this study, we characterized, identified, and determined the effect of salt-tolerant PGPR isolated from coastal saline areas on rice growth and yield. A total of 44 bacterial strains were isolated, and 5 were found to be tolerant at high salt concentration. These isolates were further characterized for salinity tolerance and beneficial traits through a series of quantitative tests. Biochemical characterization showed that bacterial survivability decreases gradually with the increase of salt concentration. One of the strains, UPMRB9, produced the highest amount of exopolysaccharides when exposed to 1.5M of NaCl. Moreover, UPMRB9 absorbed the highest amount of sodium from the 1.5M of NaCl-amended media. The highest floc yield and biofilm were produced by UPMRE6 and UPMRB9 respectively, at 1M of NaCl concentration. The SEM observation confirmed the EPS production of UPMRB9 and UPMRE6 at 1.5M of NaCl concentration. These two isolates were identified as *Bacillus tequilensis* and *Bacillus aryabhattai* based on the 16S rRNA gene sequence. The functional group characterization of EPS showed the presence of hydroxyl, carboxyl, and amino groups. This corresponded to the presence of carbohydrates and proteins in the EPS and glucose was identified as the major type of carbohydrate. The functional groups of EPS can help to bind and chelate Na^+^ in the soil and thereby reduces the plant’s exposure to the ion under saline conditions. The plant inoculation study revealed significant beneficial effects of bacterial inoculation on photosynthesis, transpiration, and stomatal conductance of the plant which leads to a higher yield. The *Bacillus tequilensis* and *Bacillus aryabhattai* strains showed good potential as PGPR for salinity mitigation practice for coastal rice cultivation.

## Introduction

Rice is considered as a significant food for the people, and in Malaysia, the self-sufficiency level is only around 75% with an average yield of 4.5 t ha^-1^ per season. To counter the ever-increasing demand, rice production needs to be increased by approximately 7 t ha^-1^ per season [1]. However, the cropping systems based on rice are vulnerable to climate change. Huge portions of rice-cultivated land are situated in risky regions, mostly in the coastline areas of South and Southeast Asia, which fulfils more than 65% of global rice demand [2]. Climate change leads to the rise of seawater level, which causes flood and triggers the intrusion of saltwater into the inland areas. It is reported that more than 50% of arable land will be threatened by 2050 due to the effect of soil salinization which is the consequence of climate change, improper irrigation practices, excess application of chemical fertilizers, and lack proper drainage systems [3]. It is also predicted that by 2056 nearly 100,000 ha of Malaysian rice-growing areas will be affected by salt stress [4]. Salinity destructively interrupts the physical and chemical properties of soil as well as affects crop growth to a higher extent [5].

To mitigate this situation, beneficial microorganisms known as plant growth-promoting rhizobacteria (PGPR) could play an important role. This group of rhizospheric bacteria could effectively colonize plant roots and maintains soil fertility by offering a favourable alternative to inorganic fertilizers and pesticides [6]. The effectiveness of PGPR to increase the growth of various crops under salt stress conditions have been reported previously [7,8]. The preliminary selection of locally-isolated salt-tolerant PGPR for salinity mitigation is crucial to ensure the effectiveness, and it has been reported that the indigenous strains are more efficient in boosting plant resistance to salinity stress compared to PGPR originated from the non-saline ecosystem [9,10]. These beneficial microbes possess several mechanisms for salt stress mitigation such as by retaining appropriate Na^+^/K^+^ ratio through secretion of extracellular polymeric substances called exopolysaccharide (EPS) that ensures their survivability under unfavourable soil conditions [11,12]. Exopolysaccharides are also essential for bacterial aggregation or flocculation yield production, which can be explained as specific adsorption of the polymeric segment and polymer bridging between cells [13]. Besides, EPS are effectively useful in constructing bacterial biofilm and enriching bacterial association on plant root surfaces [14]. Previous findings have reported that several bacterial genera, including *Pseudomonas*, *Bacillus*, *Burkholderia*, *Enterobacter*, *Microbacterium*, *Planococcus*, *Halomonas* could produce EPS in salt stress condition [15,16]. In addition to that, PGPR are able to produce multiple plant growth-promoting properties such as indole acetic acid production, biological nitrogen fixation, solubilization of soil phosphorus (P) and potassium (K), and production of siderophores and hydrolyzing enzymes under salt stress condition [17,18].

The association between PGPR and plants has long been established. Recent reviews have also revealed some possible mechanisms of PGPR for salinity tolerance by acting as endophytes and serving the plant host with the plant growth-promoting characteristics, along with improving osmolytes, anti-oxidant, and phytohormonal signalling and enhancing plant nutrient uptake efficiency [19,20]. However, there are still other mechanisms to explore as not all PGPR can enter the plant host as endophytes. The potential for the application of salt-tolerant PGPR to increase rice production in Malaysia is high, especially in the coastal region, which is exposed to saltwater intrusion. We hypothesize that the isolation and selection of suitable local and indigenous bacterial strains to alleviate salt stress in rice plants through its multiple beneficial properties could be a climate-smart agricultural practice. Thus, the present study was conducted to characterize, identify, and determine the effect of salt-tolerant PGPR isolated from the saline rice field in Malaysia on crop growth and yield.

## Materials and methods

### Collection of rhizospheric samples

Rice roots with adhered soil were collected from seven salt-affected rice fields at Telok Che Lela (5^0^41’20.2"N 100^0^25’44.9"E) and Jilid8 (5^0^ 42’48.7"N 100^0^21’54.4"E), Mukim Bujang, Kuala Muda, Kedah, Malaysia. Official permission from Kuala Muda District Agriculture Office, Sungai Petani, Kedah was obtained before the visit.

Three sampling sites from each location were chosen based on the level of salinity (electrical conductivity) and categorized as less affected, moderately affected, and severely affected. After collection, samples were preserved inside the icebox, and subsequent analyses were undertaken at Soil Microbiology Laboratory, Department of Land Management, Faculty of Agriculture, Universiti Putra Malaysia. The electrical conductivity (EC)(1:2 soil water extract method) and the pH of the soil were measured using the standard method [21].

### Isolation of salt-tolerant rhizospheric bacteria

Rice roots were shaken vigorously to remove the non-rhizosphere soil, and the sample with adhered soil was transferred into a conical flask containing 99 mL of sterilized distilled water and shaken for 15 min. The samples were diluted through a series of 10-fold dilution, and 0.1 mL of the solution was spread on Tryptic Soy Agar (TSA) media plates amended with 0.0, 0.5, 1.0, 1.5, and 2.0 M of NaCl, respectively, and incubated at 33 °C for 24 h [22]. Bacterial colonies were chosen based on their colony morphology and preserved in TSA slant at 4°C till further use.

### Plant growth-promoting traits of bacterial isolates

The salt-tolerant strains were subjected to a series of plant growth-promoting characterizations namely the ability to fix atmospheric nitrogen, solubilize phosphate and potassium and produce indole-3-acetic acid (IAA) phytohormone following the standard protocols with modifications of the media saline level using NaCl [23]. IAA production was measured using the colorimetric method.

### Salinity tolerance characteristics of bacterial isolates

The bacterial strains were characterized for salinity tolerance traits as follows:

#### Bacterial survivability at different levels of salinity

The isolated strains were grown on agar media with different levels of salinity [24]. Approximately 20 μL of fully-grown bacterial culture were inoculated into tryptic soy broth (TSB) medium adjusted with 0.0, 0.5, 1.0, 1.5, and 2.0 M of NaCl and allowed for constant agitation for 24 h. The bacterial population of the samples was measured using a series of 10-fold dilution and total plate count (TPC) technique.

#### Exopolysaccharide production

Bacterial EPS production were measured by inoculating fully-grown bacterial culture (>7 Log_10_ CFU mL^-1^) into TSB medium adjusted with different concentrations of NaCl (0.0, 0.5, 1.0, 1.5, 2.0 M) and allowed to grow on a shaking incubator (DS-310RL, Dasol, Korea) at 30 °C and 150 rpm for 72 h. Exopolysaccharide were extracted by centrifugation of bacterial cultures at 10000 rpm for 15 min at 4°C. Pre-chilled ethanol (95%) were used to precipitate the EPS from the collected supernatant (3:1 vol:vol). The precipitated EPS were further centrifuged for 20 min at 15000 rpm. To minimize the error, the samples were dried at 58 °C for 24 h in the same centrifuge tube, and the dry weight of EPS were calculated [25]

#### Estimation of bacterial flocculation

Bacterial flocculation were estimated following the standard protocols and were expressed as floc yield [26]. Bacterial cultures were grown in TSB media at 30 °C for 72 h, and flocculations were collected using filter paper (Whatman No. 1) and oven-dried at 60 °C. The dry weight representing floc yield measurement were recorded after 2 h.

#### Biofilm formation

The biofilm formation were estimated using the microtitre plate-based protocol with some minor modifications [27]. The selected bacterial isolates were grown in a salt-amended TSB medium at 30 °C for 24 h. The bacterial cells were harvested and resuspended before adjusted to an optical density (OD) of 0.3 using UV-spectrophotometer (UV-1601, 600 Shimadzu, Japan). Approximately 200 μL of the bacterial suspension were transferred into the flat-bottomed 96-well microtitre plate and incubated for 72 h at 30 °C, without shaking. The growth medium was removed from the wells, and the biofilms exhibited on the microtiter plate’s wall were coloured with 0.01% crystal violet for 20 min. The stained bacteria biofilm was then dissolved in 200 μL of 95% ethanol and were extracted and quantified at the wavelength of 590 nm.

#### Bacterial sodium uptake

Bacterial sodium uptake were measured according to the standard protocol with some amendments [22]. The bacterial isolates were grown overnight (24 h) at 37°C in a salt-amended TSB medium and harvested by centrifugation. The harvested bacterial pellets were washed with sterile distilled water and digested overnight with 0.1 N HCl at room temperature. Supernatants were collected, and the bacterial sodium uptake were measured using a flame photometer.

### Bacterial identification using 16S rRNA gene sequence

The selected bacterial strains were identified by partial sequencing of the 16S rRNA gene [24]. A fragment of the 16S rRNA gene from the total genomic DNA were amplified by polymerase chain reaction (PCR) using universal primer, P1 (5´- GAGTTTGATCCTGCTCAG-3´) and P6 (5´GTTACCTTGTTACGACTT-3´) (BioSune Biotechnology Co. Ltd., China). The PCR product was purified using Gel/PCR DNA Mini Kit (Real Biotech, Taiwan) and sent for sequencing (First Base Laboratories Pvt. Ltd., Selangor, Malaysia). The sequence data were aligned and analyzed to identify the closest neighbours using BLAST (NCBI, USA).

### Bacterial cell observation through Scanning Electron Microscopy (SEM) under salt stress condition

One loopful of fully-grown bacterial cultures were inoculated into TSB medium amended with NaCl (1.5M) and incubated for 72 h. The bacterial pellets were collected by centrifugation at 5000 rpm for 10 min. The collected bacterial cells were fixed using 2.5% glutaraldehyde for 4–6 h at 4 °C. The samples were centrifuged, and the separated bacterial cells were washed 3 times for 10 min using 0.1M sodium cacodylate buffer. The postfixation of samples were done for 2 h at 4 °C using 1% osmium tetroxide. The bacterial cells were then washed 3 times for 10 min using 0.1M sodium cacodylate buffer. After a series of dehydration using acetone (35, 50, 75, 95, and 100%), the cell suspension were pipetted onto the aluminium foil (1 cm diameter) coated with albumin. The specimens were shifted into a specimen basket and kept in the critical dryer for 1.5 h. The samples were fixed by sticking onto the stub using double-sided tape or colloidal silver then sputter-coated in gold and viewed under SEM.

### Functional group characterization of EPS using Fourier Transform Infrared Spectroscopy (FTIR)

The EPS were extracted from the selected bacterial isolates and observed for their functional group characteristics using Fourier Transformed-Infrared spectroscopy (FT-IR), Thermo Nicolet, Avatar 370 spectrometer [40]. Pellets for infrared analysis were obtained by mixing 2 mg of dried EPS with 200 mg of KBr, and the spectrum was corrected for KBr background. The pellets were then scanned in the range of 4000–5000 cm^−1^, and the spectra were traced with a Hewlett Packard plotter (Palo Alto, CA, USA).

### Estimation of sugar composition of bacterial EPS using UPLC-RID

Approximately 1.0–2.5 g of the dried EPS synthesized by the selected bacterial isolates were dissolved in 25 mL of Ultra Performance Liquid Chromatography (UPLC) grade water, and the solution were centrifuged on a Remi C 30 centrifuge (Mumbai, India) at 16,000 rpm for 10 min and the collected supernatant were filtered through a 0.2 μm nylon filter (Micro-Por Minigen Syringe Filter, Genetix Biotech Asia, New Delhi). Sugars were analyzed using the UPLC system (Waters Acquity H-Class) on a reverse-phase Hypersil Gold Amino column (100 (L) x 2.1mm (Diam.), Particle Size: 1.9μm), using a refractive index detector (RID-10A, Shimadzu Corporation, Kyoto, Japan) with an isocratic mobile phase of ACN: H2O (80:20, v/v), maintained at a flow rate 0.6 mL min^-1^.

### Plant inoculation test under glasshouse conditions

#### Soil preparation and fertilizers application

The soils (Bernam series soil) were collected from Sawah Sempadan, block D, Tanjong Karang, Selangor, Malaysia with a pH of 4.23. The initial soil nutrient content was 0.62% of N, 0.08% of P, 0.29% of K, 0.23% of Ca, and 0.33% of Mg. Air-dried soil was sieved through a 2 mm sieve, autoclaved and 20 Kg were transferred into a plastic pot. Chemical fertilizers consisting of urea, triple super phosphate (TSP) and muriate of potash (MOP) were applied at the rate of 170-80-150 Kg ha^-1^ based on the recommendations by the Department of Agriculture, Kuala Selangor, Selangor, Malaysia.

#### Preparation of bacterial inocula and inoculation of the rice plants

The selected salt-tolerant PGPR strains were inoculated to three rice varieties, namely BRRI dha67, Putra-1, and MR297, which were previously identified as tolerant, moderately tolerant, and susceptible at 8 dSm^-1^ of salinity. The fully-grown bacterial cultures were inoculated into TSB medium and shaken for 24 h. The bacterial suspension consisting of approximately 10^8^ to 10^9^ CFU mL^-1^ were inoculated onto 5 days old rice seedlings and allowed to settle for 1h and transplanted into the plastic pot. Rice seedlings for the non-inoculated control were treated with 2 mL of sterile distilled water.

#### Soil salinity adjustment

The salinity level of the soil were adjusted using sodium chloride (NaCl) to achieve the electrical conductivity (EC) of 8 dSm^-1^. The EC level was maintained throughout the experimental period.

#### Determination of photosynthesis, transpiration and stomatal conductance

The rates of photosynthesis, transpiration, and stomatal conductance were measured at the flowering stage for each rice varieties using LI-COR (LI-6400XT) portable photosynthesis system, LI-COR-Inc Lincoln, Nebraska, USA.

#### Plant tissue analysis

Plant tissue samples were oven-dried at 70°C for 72 h and were ground using a plant grinder, and the concentration of the nutrients were determined using the standard protocol [28].

#### Measurement of yield data components

The yield data components namely filled grains percentage, 1000 grain weight, and grain yield were recorded at the terminal harvest. The rice grains were allowed to air-dry for one week before counting and weighing.

### Statistical analysis

The glasshouse experiment was conducted in a factorial completely randomized design (CRD). The collected data were analyzed following Analysis of Variance (ANOVA) using SAS 9.4 software. Means were compared by Tukey (HSD) at a probability level of 0.05.

## Results

### Determination of pH, electrical conductivity and number of bacterial isolates from saline rice areas

Forty-four rhizobacterial strains were isolated from seven sampling points at three different locations. The pH and electrical conductivity of the soil range between 3.91–6.27 and 0.41–17.64 dSm^-1^, respectively. The number of bacteria isolates were higher from the less-affected areas (S1 Table).

### Selection of salt-tolerant bacterial isolates

The number of bacterial isolates that could survive under saline agar media decreased with the increased salinity level. A total of 38, 26, 18 and 5 isolates could survive under NaCl concentrations of 0.5, 1.0, 1.5, and 2.0 M, respectively. Five isolates were able to grow at 2.0 M of NaCl and were selected as highly salt-tolerant strains and labelled as UPMRA4, UPMRB9, UPMRE3, UPMRE6, and UPMRG1.

### Plant growth-promoting properties of selected bacterial isolates

Among the five selected bacterial isolates, UPMRB9 had the highest IAA production across all salinity levels and the values consistently reduced with increased salinity levels which ranged from 5.91 to 21.18 μg IAA mL^-1^ (S2 Table). All bacterial isolates were able to fix atmospheric nitrogen (except for UPMRA4) at lower salinity levels (0.0–1.0 M), and only UPMRB9 and UPMRE6 showed nitrogen fixation activities at maximum salinity level of 2.0 M NaCl. UPMRE6 also showed phosphate and potassium solubilization activities at the maximum salinity level.

### Effect of NaCl concentrations on salinity tolerance traits of selected bacterial isolates

#### Bacterial population

The bacterial population were recorded by measuring the number of colony-forming units (CFU) in the culture media plate. All bacterial isolates showed optimum growth at 0 M of NaCl, followed by a gradual decline of the population with the increase of salinity level (Table 1). At 2.0 M of NaCl, UPMRB9 and UPMRG1 showed significantly higher population as compared to the other three strains.

**Table 1:** Effect of NaCl concentrations on bacterial population (Log_10_ CFU mL^-1^).

Bacterial isolate	0.0 M	0.5 M	1.0 M	1.5 M	2.0 M
UPMRA4	10.20c	8.68c	6.59b	5.53c	4.16bc
UPMRB9	11.26a	10.87a	9.49a	8.78a	5.56a
UPMRE3	8.82e	6.73d	5.83b	4.66c	3.77c
UPMRE6	9.03d	8.34c	8.34a	7.32b	4.88ab
UPMRG1	10.90b	9.87b	9.17a	8.53a	5.32a
Mean	10.04	8.90	7.88	6.96	4.74
HSD_(0.05)_	0.14	0.99	1.58	1.12	0.82

Different letters indicate significantly different means (Tukey HSD post-hoc test, p<0.05)

#### Exopolysaccharide production

**Fig 1:**
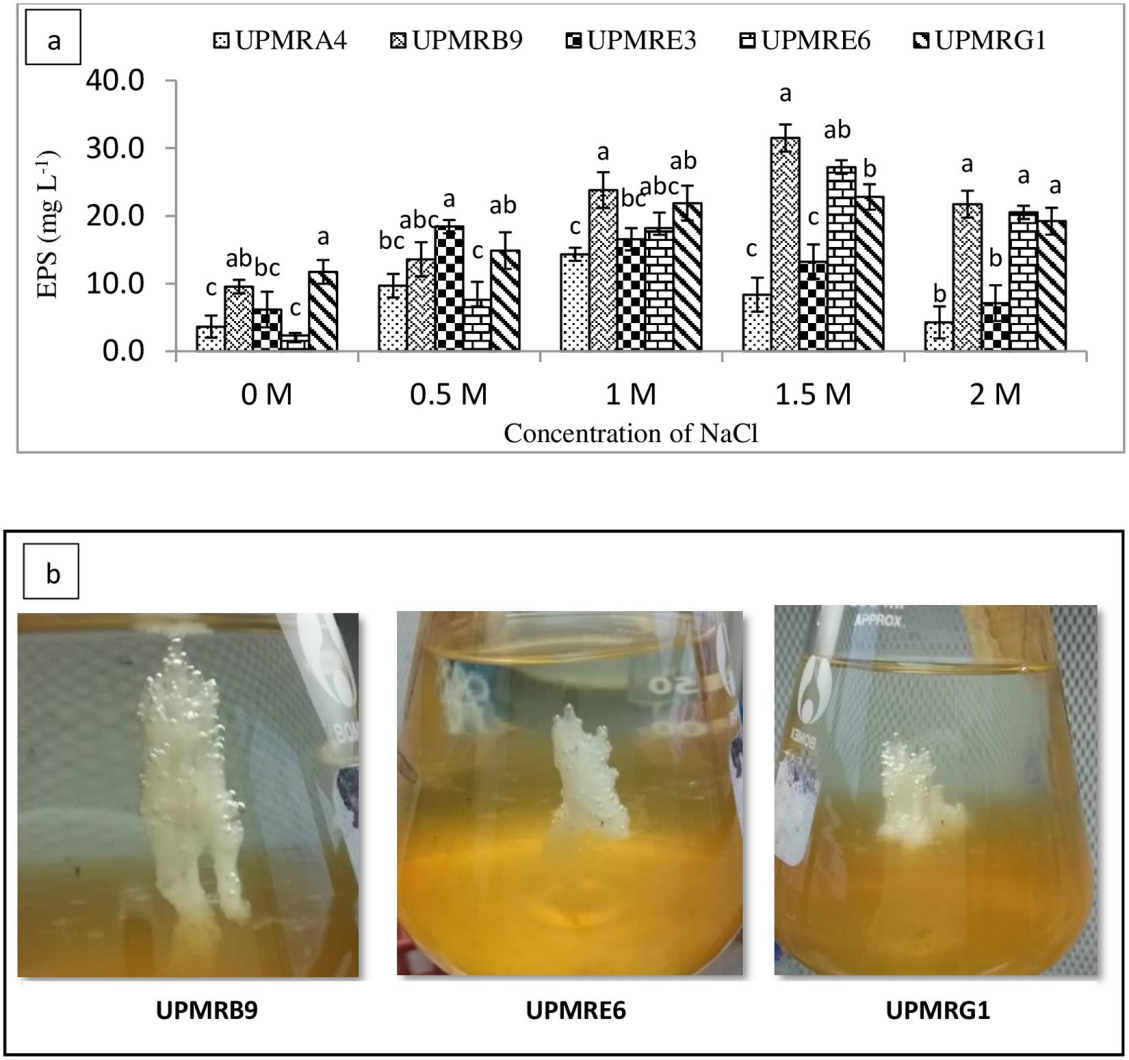
a. Effect of NaCl concentration on bacterial exopolysaccharide production. Means having the same letter within each concentration do not differ significantly at the probability level 0.05 by Tukey (HSD). b. Exopolysaccharides production of the bacterial isolates in 1.5M of NaCl-amended TSB medium.

The bacterial EPS production were significantly affected by the salinity level of the medium (Fig 1a and 1b). The significantly highest EPS production were recorded by UPMRB9 (31.50 g L^-1^) at 1.5M of NaCl concentration. Results showed that at the highest NaCl concentration, three isolates, namely UPMRB9, UPMRE6, and UPMRG1, produced significantly higher EPS compared to UPMRA4 and UPMRE3.

#### Bacterial flocculation yield

**Fig 2:**
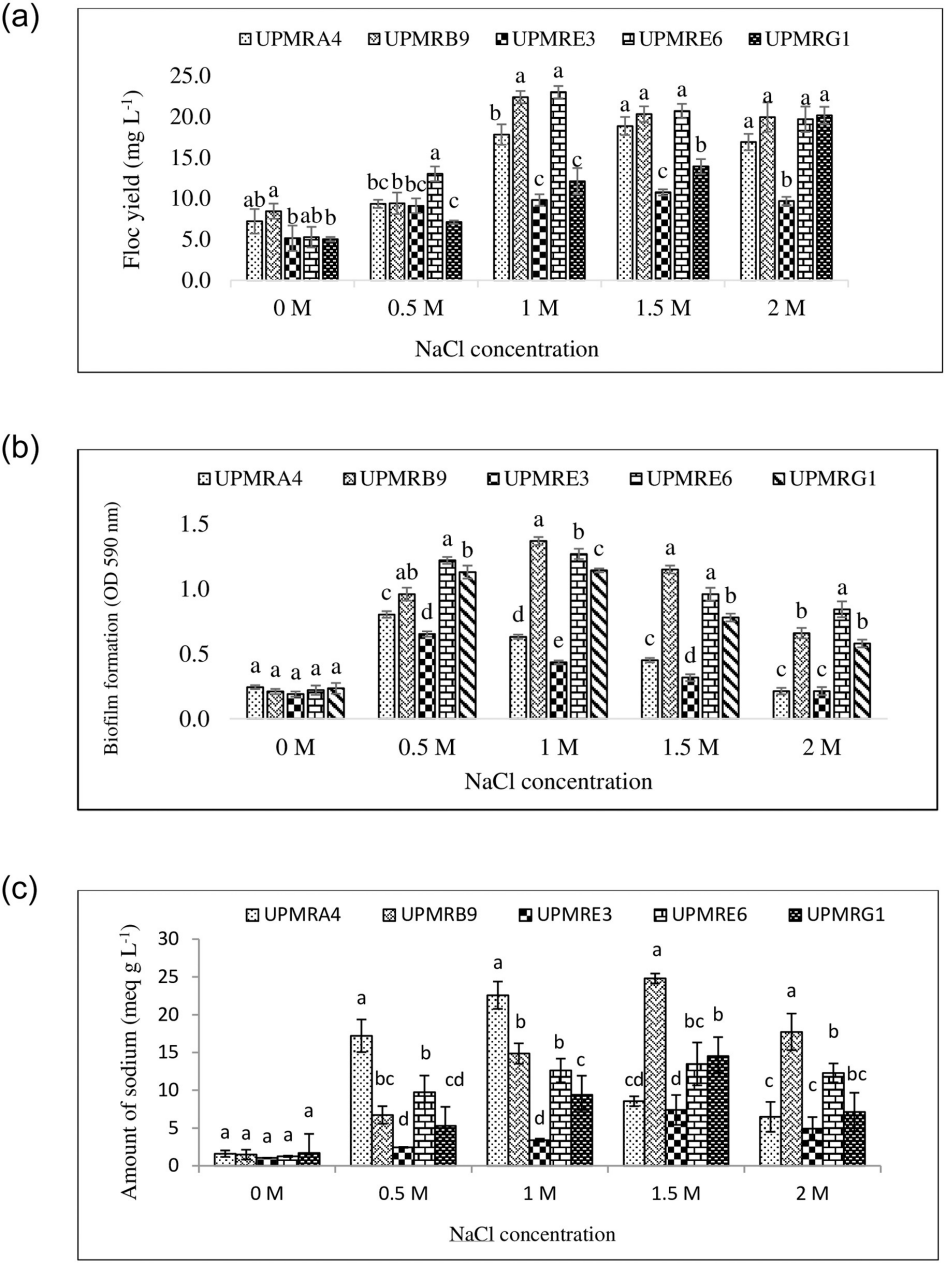
a. Effect of NaCl concentration on bacterial floc yield production. Means having the same letter within each concentration do not differ significantly at alpha 0.05 by Tukey (HSD). b. Effect of NaCl concentration on bacterial biofilm formation. Means having the same letter within each concentration do not differ significantly at the probability level 0.05 by Tukey (HSD). c. Effect of NaCl concentration on bacterial uptake of sodium. Means having the same letter within each concentration do not differ significantly at alpha 0.05 level by Tukey (HSD).

Bacterial flocculation yield increased gradually with the increase of NaCl concentration (Fig 2a). Significantly highest flocculation yield were recorded by UPMRE6 and UPMRB9 at 1.0 M of NaCl. The yield production of all isolates decreased slightly at 1.5 M and 2.0 M of NaCl concentrations.

#### Bacterial biofilm formation

The biofilm formation is closely associated with EPS production. Maximum biofilm formation for all bacterial isolates were recorded at 1.0 M of NaCl, followed by a gradual decline at 1.5M and 2.0 M of NaCl (Fig 2b).

#### Bacterial uptake of sodium

The bacterial sodium uptake at different NaCl concentrations is presented in Fig 2c. The significantly highest sodium uptake was recorded at 1.5M of NaCl by UPMRB9 with the values of 24.8 meq L^-1^.

#### SEM observation

**Fig 3:**
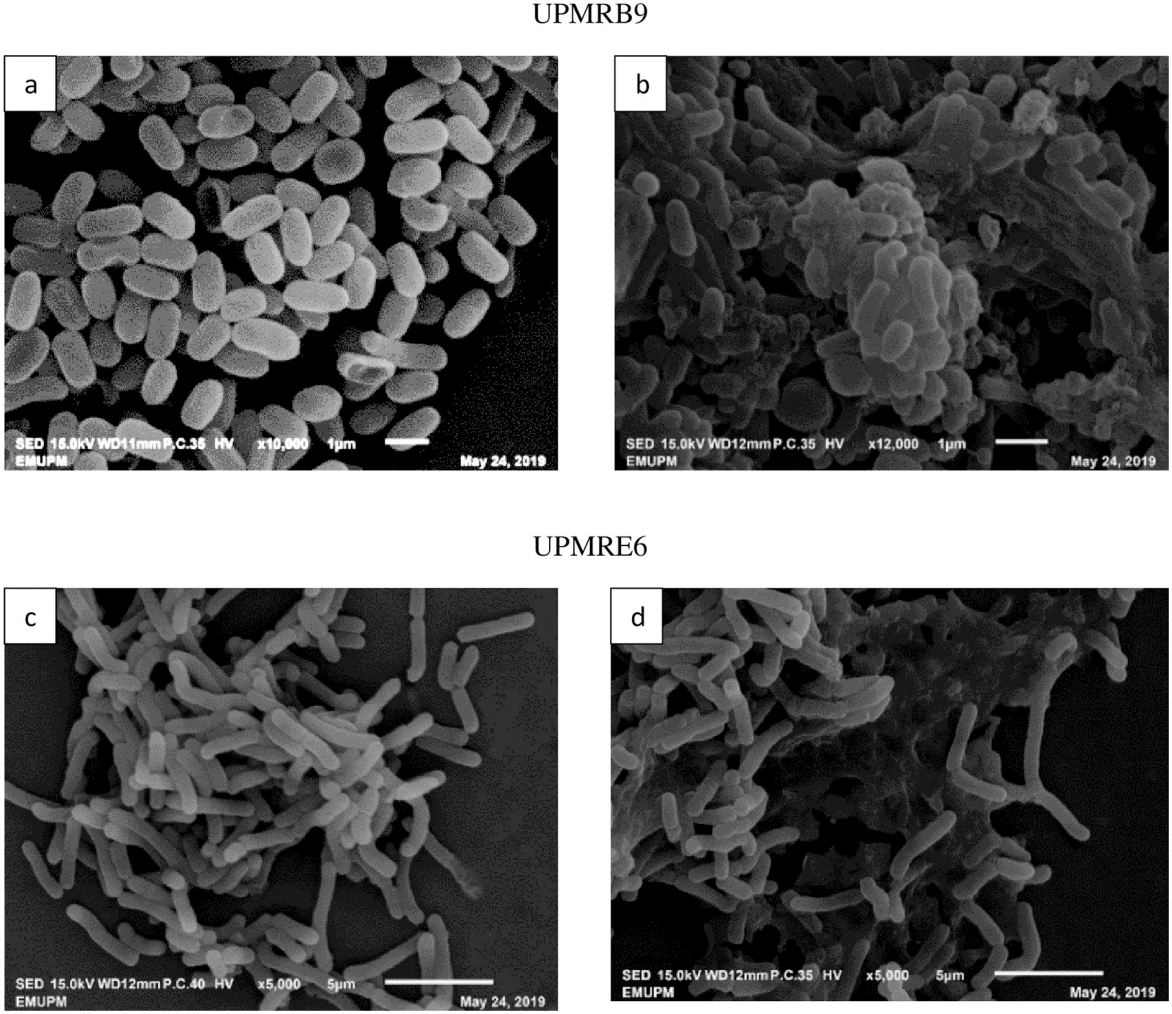
SEM images showing the cells of UPMRB9 and UPMRE6 in (a, c) non-saline and (b, d) saline conditions.

Scanning electron microscopy showed the presence of EPS produced by UPMRB9 and UPMRE6 under saline conditions. Under the non-saline condition, the cells of UPMRB9 were in scattered position (Fig 3a) while under the saline condition, the cells started to form aggregation through EPS production (Fig 3b). Similar findings were observed with UPMRE6 in which the strain produce EPS leading to aggregate formation under the saline condition as compared to non-saline condition (Fig 3c and 3d).

#### Identification of selected isolates based on partial 16S rRNA gene sequences

Two bacterial strains (UPMRB9 and UPMRE6) were selected for molecular identification based on their optimum salt tolerance and plant growth-promoting characteristics. The 16S rRNA fragments were successfully amplified using Polymerase Chain Reaction (PCR). Approximately 1413bp and 1411bp were sequenced for UPMRB9 and UPMRE6, respectively. The BLASTX comparison searches against the NCBI nucleotide database revealed 99.0% similarity of the isolates UPMRB9 with *Bacillus tequilensis*10b (NCBI accession number: NR 104919.1) and UPMRE6 with *Bacillus aryabhattai*B8W22 (NCBI accession number: NR 115953.1) The phylogenetic analysis of these isolates was also done based on neighbour-joining bootstrap analysis (S1 Fig).

#### Functional group characterization of bacterial EPS using FT-IR spectra

The functional group of EPS produced by the selected isolates were analysed, and the broad peak of EPS samples was at 3400 cm^-1^ (3200–3600) which is specified for O-H symmetric stretch vibration in polymeric compounds (S2 Fig). Moreover, the peaks at 3367.71 cm^−1^, 1630–1660 cm^−1^, 1400–1420 cm^−1^, 1,050–1150 cm^−1^ and 1080.14 cm^−1^ were ascribed to N–H stretching vibrations of protein and C–H stretching vibrations, C = O and C-N symmetric stretch vibration (peptidic bond of proteins corresponding to the group of amides I), stretching vibration of C = O in carboxylates, C-O stretching vibrations with an alcoholic functional group and O-H symmetric stretch vibration in polysaccharides and their derivatives, respectively.

The peaks at <1,000 comprised of several functional groups such as phosphate and sulfur.

#### Sugar composition analysis of bacterial EPS using UPLC

The sugar composition of the EPS extracted from the two bacterial isolates were analyzed using UPLC. The chromatographic peaks only showed the presence of glucose in the EPS sample (S3 Fig).

### Plant inoculation test under glasshouse conditions

#### Effect of salt-tolerant PGPR inoculation on photosynthesis, transpiration and stomatal conductance of three rice varieties under saline condition

**Fig 4:**
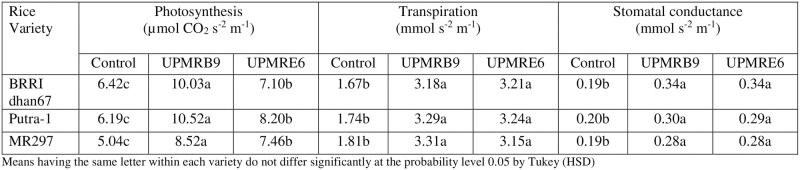
Effect of bacterial inoculation on the rate of photosynthesis, transpiration and stomatal conductance of three rice varieties under saline condition. Means having the same letter within each variety do not differ significantly at the probability level 0.05 by Tukey (HSD).

Under salt stress conditions, the inoculation of the selected PGPR significantly increased the rate of photosynthesis, transpiration, and stomatal conductance for all rice varieties, compared to the uninoculated plants (Fig 4). Inoculation of UPMRB9 to BRRI dhan67, Putra-1, and MR297 causes an increment of photosynthesis rate by 56.23%, 69.95%, and 69.04%, respectively. The transpiration rate of BRRI dhan67 increased by 92.22% by UPMRE6 inoculation, and UPMRB9 increases the transpiration rates of Putra-1 and MR297 by 89.08% and 82.87%, respectively.

#### Effect of bacterial inoculation on nutrient uptake of three rice varieties under saline condition

The inoculations of UPMRB9 and UPMRE6 to three rice varieties causes a significant increased of the nutrients uptake, under saline condition (S3 Table). The increase ranges from 39.88% to 276.47%, depending on the nutrients. The highest increment was on phosphate uptake of rice variety BRRI dhan67 inoculated with UPMRE6.

#### Effect of bacterial inoculation on yield components of three rice varieties under saline condition

Bacterial inoculations cause a significant increment of the yield components for all rice varieties (Table 2). UPMRB9 inoculation causes greater improvement of the filled grains, 1000 grain weight and grain yield of the three rice varieties by up to 15.44%, 33.12% and 57.55%, respectively.

**Table 2:** Effect of bacterial inoculation on yield data components of three rice varieties under saline condition.

Variety	Filled grains %			1000 grain weight			Grains plant^-1^ (g)		
	Control	UPMRB9	UPMRE6	Control	UPMRB9	UPMRE6	Control	UPMRB9	UPMRE6
BRRI dhan67	62b	68.67a	66.33ab	20.53b	27.33a	22.80b	20.16b	27.66a	25.47a
Putra-1	52a	57.33a	54.33a	17b	21.43a	19.43a	17.81b	24.48a	23.26a
MR297	45.33b	52.33a	48.67a	19.67b	24.13a	20.67ab	14.37b	22.64a	21.37ab

Means having the same letter within each variety do not differ significantly at the probability level 0.05 by Tukey (HSD)

## Discussion

Salinity is one of the soil stress which hampered the rice production in the coastal area. Therefore, efforts to mitigate this problem have been made, including the use of salt-tolerant PGPR as in this study. Five bacterial strains were selected from the initial forty-four isolates, based on their survivability under high salt condition. These strains exhibit multiple plant growth-promoting characters, namely ability to produce IAA, fix atmospheric nitrogen and solubilize phosphate and potassium on saline media. The plant growth-promoting properties decreased gradually with increased salt concentration. Salt-tolerant bacterial strains have been shown to have high nitrogenase activity under saline conditions and can produce osmolytes to maintain cell turgidity and metabolism under the unfavourable condition [29,30].

Various reports have indicated the possible mechanisms of salt-tolerance of PGPR particularly by acting as endophytes. However, only a fraction of the beneficial bacteria can enter the root cell, and the plant-microbe interaction mostly occurs within the 5mm rhizosphere zone. In this study, two isolates were able to maintain a considerably high bacterial population at 1.5M of NaCl-amended media, which is similar to the findings of a previous report [31]. These isolates were also able to produce higher EPS, flocculation yield and biofilm. Bacterial EPS production is an important salt-tolerant characteristic which is always associated with biofilm production. Exopolysaccharides are able to lessen the hostile effect of osmotic-stress by augmenting fresh weight, dry weight and water content in plants [32]. All these salt-tolerance characteristics were supported by the SEM observation, which showed the bacterial ability to produce EPS, flocculate and biofilm formation when exposed to the saline condition as compared to non-saline media. Bacterial cells could associate with the plant root system to significantly improve moisture holding capacity and defence system against different abiotic stresses. The reduction of bacterial EPS and biofilm formation with increased NaCl concentration have been reported by the previous researcher as well [33]. Plant growth-promoting rhizobacteria with EPS-producing characters chelate different cations including Na^+^, and it has been found that under salinity stress, bacteria can bind with the Na^+^ ion through the secretion of EPS which consequently reduces its toxicity in the soil [34]. Therefore, a higher population of EPS-producing bacteria in the rhizosphere zone is likely to reduce the concentration of available Na^+^ for plants uptake and consequently alleviates salt stress effect on plants under saline environment.

The selected PGPR isolates were identified using the 16S rRNA gene sequence, and comparison with the NCBI database revealed their closest similarity as *Bacillus tequilensis* and *Bacillus aryabhattai*. *Bacillus* is a well-known genus categorized as PGPR, and it is also one of the major genus exhibiting EPS-production ability under saline soil conditions [35]. *Bacillus tequilensis* and *Bacillus aryabhattai* have also been isolated and studied by other researchers as PGPR on various crops [36,37].

The functional group of EPS produced by the selected isolates were analyzed by using Fourier Transform infrared spectroscopy (FT-IR), and the results were found to be almost similar. The EPS are mostly structured by carboxyl, hydroxyl and amino functional groups due to the high protein and carbohydrate contents. It was reported that these functional groups of bacterial EPS could relieve salt stress in plants by binding with the Na^+^ ion [38,39]. Exopolysaccharide have unique binding properties by acting as rhizosheath, a physical barrier surrounding the roots, which will prevent Na^+^ from attaching to the roots and thereby inhibits their absorption by plants [40,41]. The sugar composition analysis of EPS secreted by the isolates revealed the presence of glucose as the main carbohydrate, which is similar to findings of various earlier reports [42,43]. It is interesting to note that other researchers have reported that the composition of EPS molecules formed by the bacteria is variable with the presence of several sugar molecules such as glucose, galactose, mannose, xylose, glucuronic and galacturonic acid [44,45]. In addition to simple sugar molecules, EPS are also composed of amino sugars (N-acetylamino sugars), uronic acids (fructose and rhamnose), neutral sugars (galacturonic), pyruvateketals and ester-linked substituents.

The glasshouse study has shown that inoculations of two salt-tolerant PGPR strains have led to a significant improvement of plant photosynthesis, transpiration, and stomatal conductance of three rice varieties. The moderately-tolerant rice variety Putra-1 recorded the highest rate of photosynthesis under saline conditions upon inoculation with *Bacillus tequilensis* UPMRB9. The inoculation of both strains also increased stomatal conductance and transpiration rate of the rice varieties. Higher bacterial IAA production could facilitate the root growth and increase the uptake of water and nutrients for the plant. It has been reported previously that the flow of water and nutrients could stabilize stomatal conductance and the rate of transpiration. Moreover, the sodium binding capacity of PGPR causes stable cellular turgidity and thereby protect the plant’s chloroplast from the damaging effect of salinity, resulting in higher chlorophyll synthesis and rate of photosynthesis and starch production, consequently, improving plant growth under salt stress conditions [46–49]. The nutrients uptake were also significantly improved by the inoculation of the selected bacterial strains. These might be attributed to the bacterial alteration of the root structure leading to the increased secretion of root exudates. The previous report has shown that PGPR colonizes plant roots and enhances root health by providing nutrients and in return, receives exudates from their associates [50].

The yield data showed that uninoculated plants were susceptible to saline stress, leading to significantly lower grain production. Excessive sodium ion will disturb the process of photosynthesis by shrinking cell organelles, reducing enlargement and differentiation of tissues, nutritional imbalance and causing cell membrane damage [51]. These adverse effects of salinity could be reduced with the application of salt-tolerant PGPR, as shown in this glasshouse trial. The inoculations of the selected isolates have significantly increased the photosynthesis of all three rice varieties, leading to higher grain yield under saline condition. Similar findings of yield increment were found upon inoculation of PGPR to various crops under salt stress conditions [52,53].

The selection criteria of beneficial microorganisms for salinity mitigation is critical to ensure its infectiveness and effectiveness to the plant. This study reports the isolation and characterization of salt-tolerant PGPR from the salt-affected area and the plant inoculation test under saline conditions. A future study involving the field trial on the affected areas is crucial to ensure that the locally-isolated strains can survive the soil condition and are compatible with the conventional farmer practices.

## Conclusion

This study reports the characterization of two locally-isolated PGPR strains identified as *Bacillus tequilensis and Bacillus aryabhattai*, which have shown salt-tolerance and plant growth-promoting characteristics under saline conditions. The plant inoculation test revealed that these strains were capable of increasing the rate of photosynthesis, transpiration and stomatal conductance of three rice varieties, consequently leading to a higher grain yield production. Given these promising potential, the *Bacillus tequilensis* and *Bacillus aryabhattai* strains would be a suitable candidate for biofertilizer practices for salinity mitigation in saline-affected coastal rice cultivation.

## Supporting information

S1 Table pH, electrical conductivity and number of bacterial isolates in soil collected from three different locations. (TIF)

S2 Table Plant growth-promoting traits of the selected salt-tolerant bacterial isolates. Different letters indicate significantly different means (Tukey HSD post-hoc test, p<0.05). ‘+’ indicates positive, ‘-’ indicates negative.
(TIF)

S3 Table Effect of bacterial inoculation on nutrient uptake of three rice varieties under saline condition. Means having the same letter within each variety do not differ significantly at the probability level 0.05 by Tukey (HSD).
(TIF)

S1 Fig Neighbour-joining tree based on 16S rRNA gene sequences showing the phylogenetic relationship of UPMRB9 and UPMRE6 to related isolates; bar represents the nucleotide divergence value. (TIF)

S2 Fig FTIR spectra of EPS extracted from the bacterial isolates (a) UPMRB9 and (b) UPMRE6. (TIF)

S3 Fig UPLC chromatogram shows the retention time of glucose in EPS sample synthesized by two bacterial isolates a) UPMRB9 and b) UPMRE6. (TIF)
