# Supplementary figures and images for: Characterization of salt-tolerant plant growth-promoting rhizobacteria and the effect on growth and yield of saline-affected rice
Source: PLoS One. 2020 Sep 4;15(9):e0238537. doi: 10.1371/journal.pone.0238537 (PMC7473536; doi:10.1371/journal.pone.0238537)

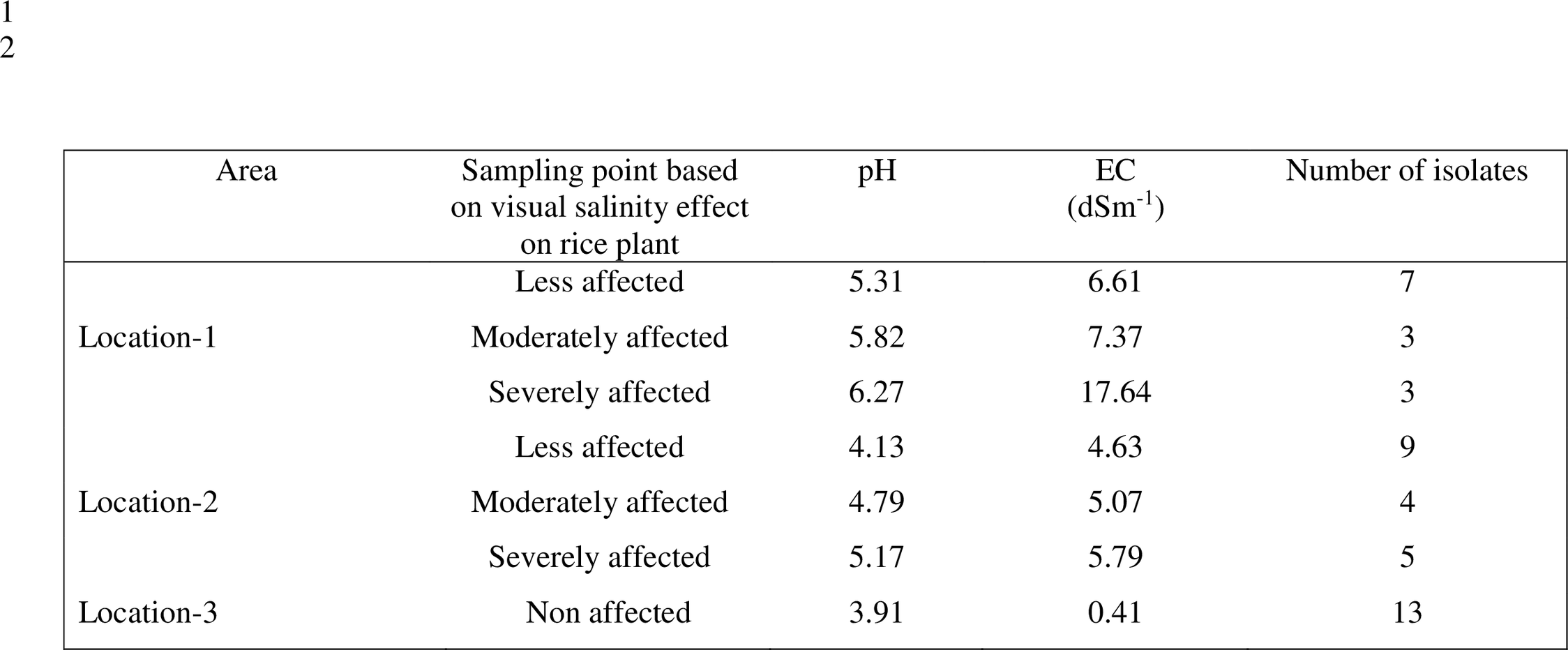

Supplement: S1 Table — (TIF) [file pone.0238537.s001.tif]

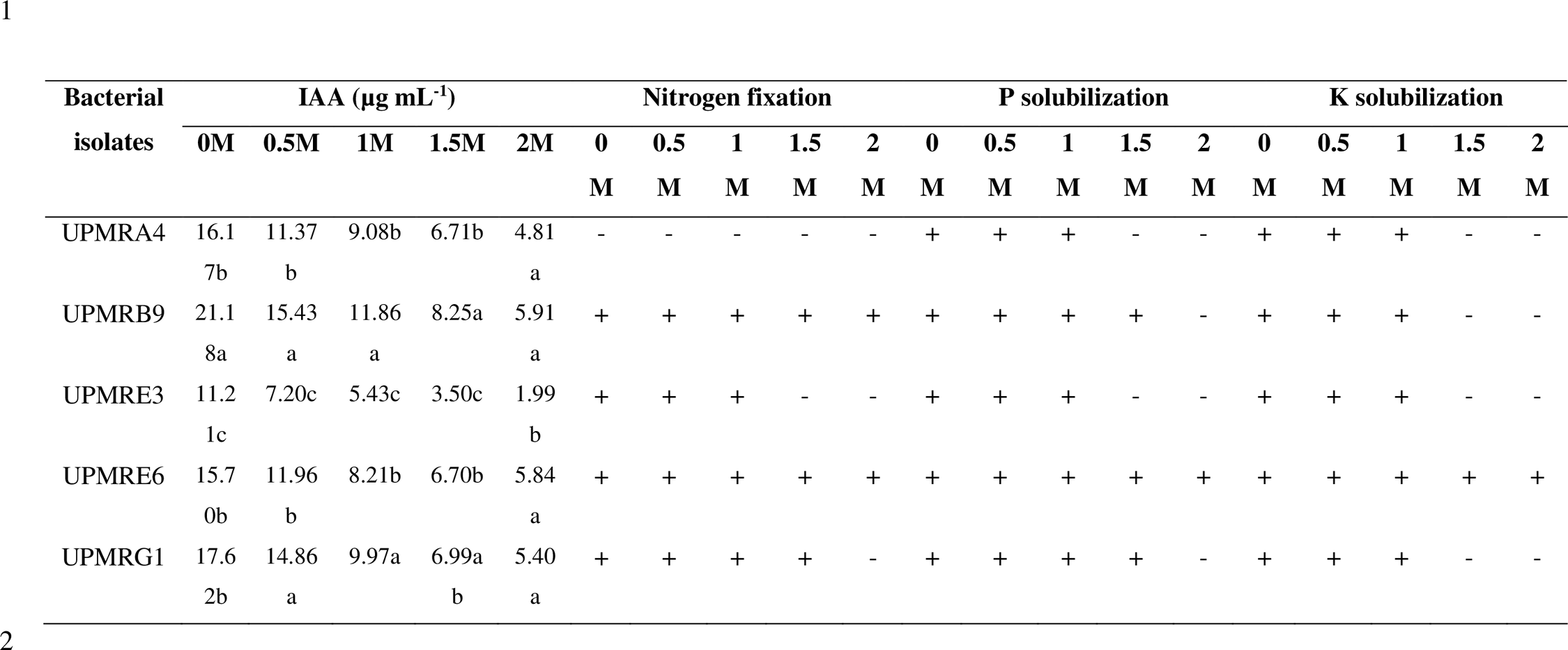

Supplement: S2 Table — Different letters indicate significantly different means (Tukey HSD post-hoc test, p<0.05). ‘+’ indicates positive, ‘-’ indicates negative. (TIF) [file pone.0238537.s002.tif]

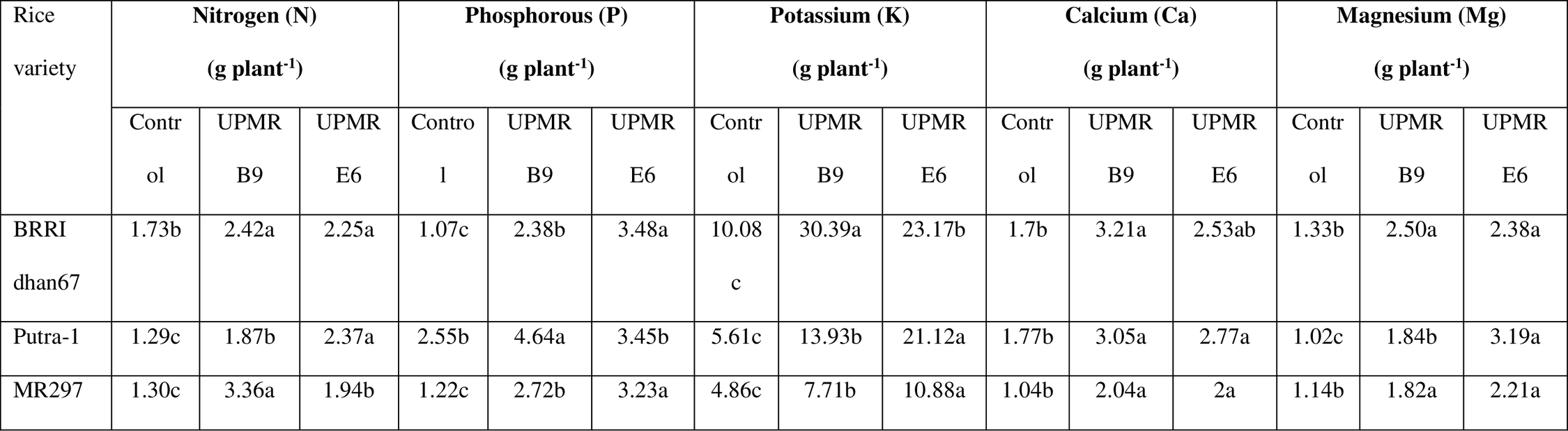

Supplement: S3 Table — Means having the same letter within each variety do not differ significantly at the probability level 0.05 by Tukey (HSD). (TIF) [file pone.0238537.s003.tif]

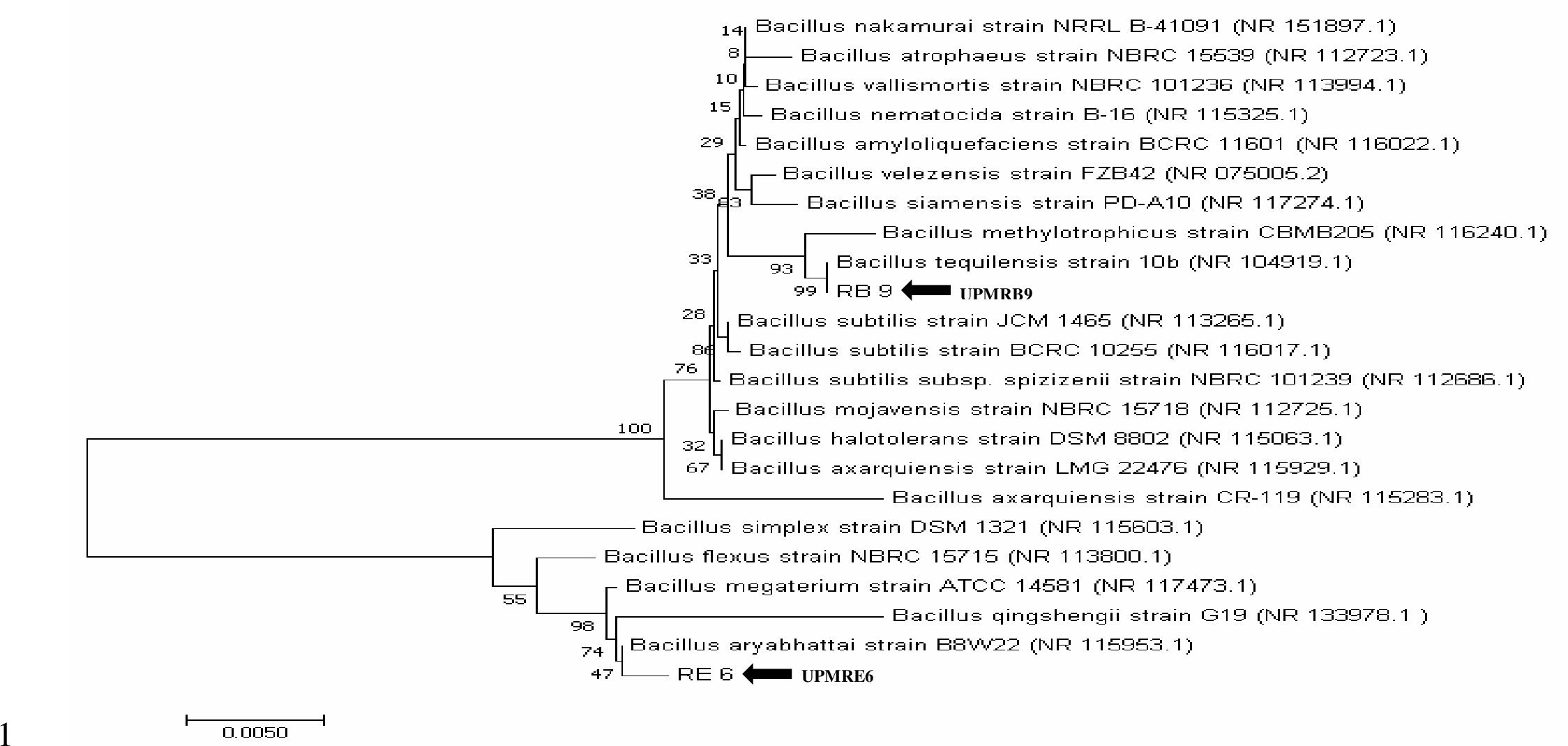

Supplement: S1 Fig — (TIF) [file pone.0238537.s004.tif]

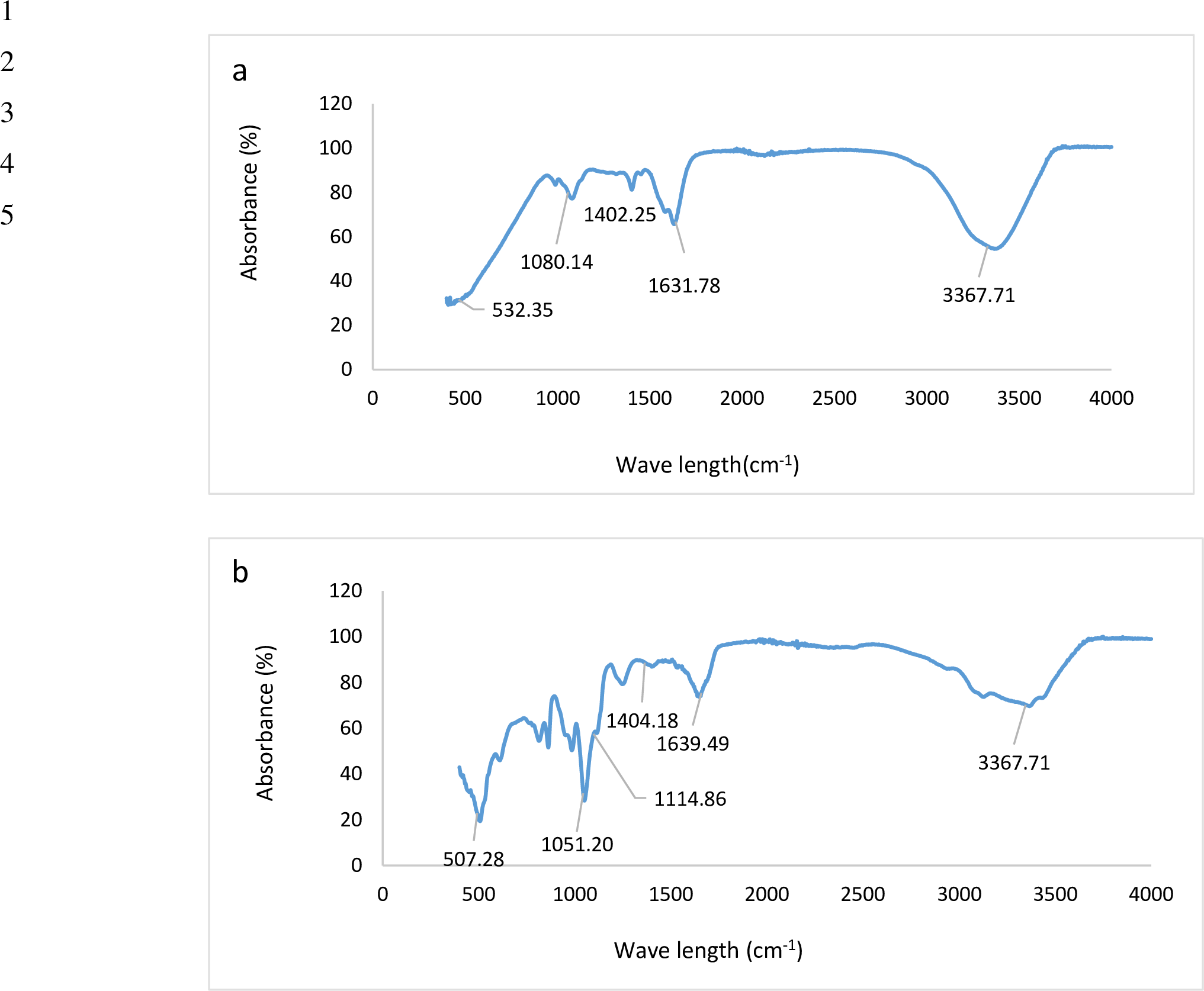

Supplement: S2 Fig — (TIF) [file pone.0238537.s005.tif]

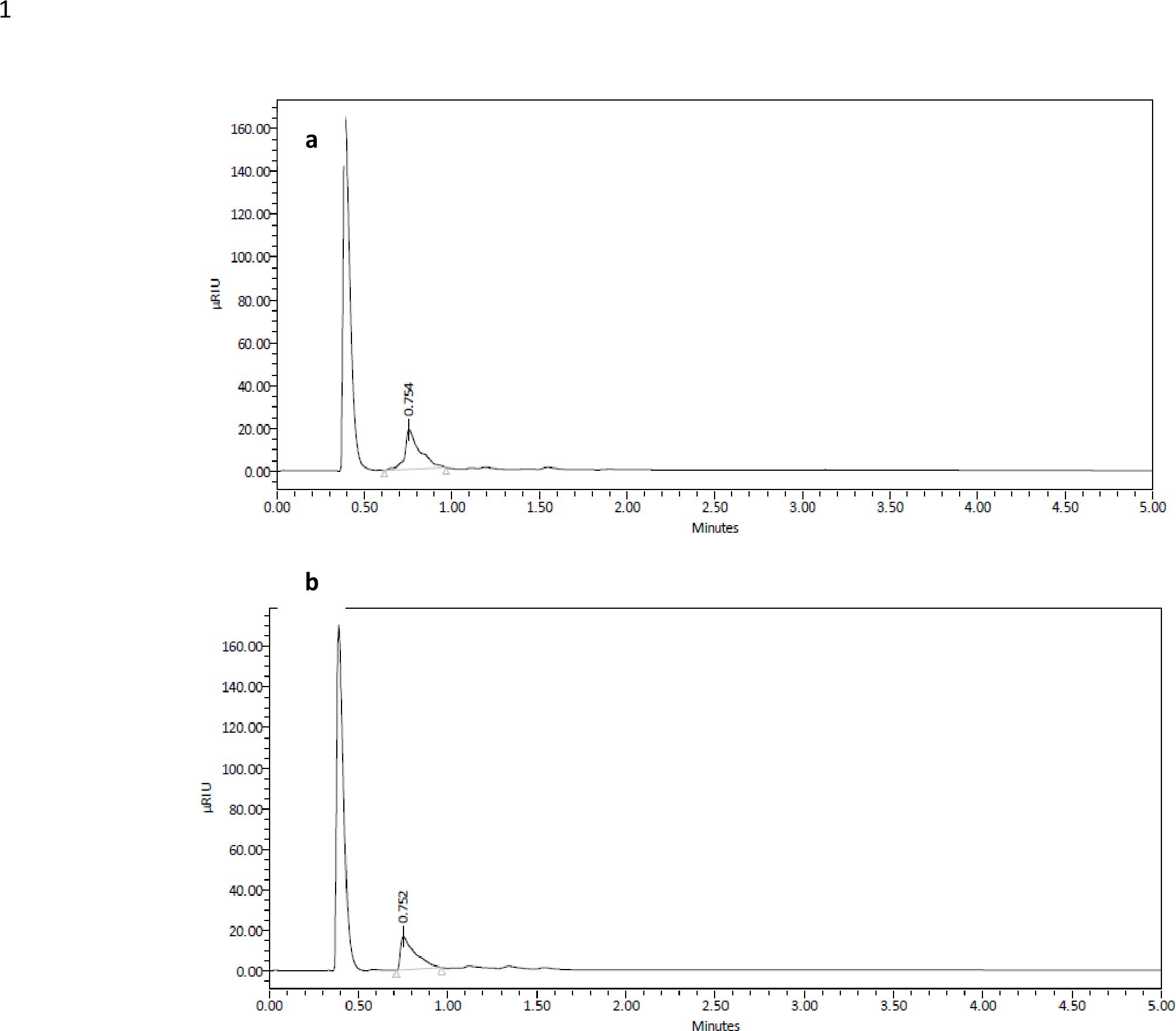

Supplement: S3 Fig — (TIF) [file pone.0238537.s006.tif]
